# A Facile Pathway to Modify Cellulose Composite Film by Reducing Wettability and Improving Barrier towards Moisture

**DOI:** 10.3390/ma10010039

**Published:** 2017-01-05

**Authors:** Xiaorong Hu, Lin Chen, Dandan Tao, Zhaocheng Ma, Shilin Liu

**Affiliations:** 1 Department of Cardiology, Renmin Hospital, Wuhan University, Wuhan 430070, China; huxrwurm@163.com; 2 Key Laboratory of Horticultural Plant Biology of Ministry of Education, College of Horticulture and Forestry Sciences, Huazhong Agricultural University, Wuhan 430070, China; linchen@webmail.hzau.edu.cn (L.C.); slliu2013@mail.hzau.edu.cn (S.L.); 3 School of Chemical and Material Engineering, Jiangnan University, Wuxi 214122, China; 13657241416@163.com

**Keywords:** polymer-matrix composites (PMCs), cellulose, wettability, curing

## Abstract

The hydrophilic property of cellulose is a key limiting factor for its wide application. Here, a novel solution impregnation pathway was developed to increase the hydrophobic properties of cellulose. When compared with the regenerated cellulose (RC), the composite films showed a decrease in water uptake ability towards water vapor, and an increase of the water contact angle from 29° to 65° with increasing resin content in the composites, with only a slight change in the transmittance. Furthermore, the Young’s modulus value increased from 3.2 GPa (RC film) to 5.1 GPa (RCBEA50 film). The results indicated that the composites had combined the advantages of cellulose and biphenyl A epoxy acrylate prepolymer (BEA) resin. The presented method has great potential for the preparation of biocomposites with improved properties. The overall results suggest that composite films can be used as high-performance packaging materials.

## 1. Introduction

To address increasing concerns over the depletion of non-renewable fossil resources, bio-based composites are being modified in various ways to extend their usage to different industries [1,2]. Cellulose, a unique biopolymer, is a raw material that is renewable and almost inexhaustible. Currently, cellulose has attracted the attention of researchers all over the world, which will greatly benefit economics and the environment. However, cellulose is difficult to process by the thermal or dissolution method due to its strong hydrogen bonding interaction. Many efforts have been made to consolidate cellulose into plastic matrixes [3,4,5]. It was well known that the properties of cellulose materials were sensitive to humid conditions, and the hydrogen bonding interactions could be destroyed, which results in a significant expansion of the amorphous regions and an increase of the chain segmental motion, finally leading to a significant decrease in the mechanical performance [6,7]. In the last few decades, cellophane, a conventional polymer, has been tarnished as a result of its high costs in production and environmental pollution (CS_2_ and H_2_S) in manufacture and post-treatment [8].

Chemical alteration of cellulose is often used to modify the performance of cellulose for specific applications. To this end, several strategies have been developed, including homogeneous [9,10,11] and heterogeneous conditions [12,13,14]. Despite the use of cellulose on a large scale, many potential applications of cellulose derivatives are limited by their hygroscopic properties because of the change in the cellulose chemical structure. Additionally, some complicated and sophisticated preparation methods were used for the functionalization of cellulose nanofibers [15,16,17,18]. In many methods, the hydrophilicity of cellulose was needed to be modified by using a polar solvent or hydrophilic matrix. However, the efficient hydrophobization of the nanofibrils still remains a great challenge.

In our previous work, we focused on the cellulose dissolution by using alkali/urea aqueous solvents [19,20]. Regenerated cellulose materials prepared from the aqueous alkali/urea (NaOH or LiOH/urea) solvent had porous structure, and the porous structure was an optimum scaffold for controllable synthesis of inorganic nanoparticles in situ. Furthermore, conductive polymer monomers could also be polymerized in the porous structured cellulose matrix, and cellulose-based conductive paper has already been obtained [21]. In the present study, inspired by these interesting results, cellulose composite films with enhanced water resistance and mechanical properties have been developed by using curable prepolymer through ultraviolet (UV) exposure. Specifically, the porous regenerated cellulose films were impregnated with biphenyl A epoxy acrylate prepolymer (BEA) solutions, and under UV-initiated curing reaction of BEA prepolymers, the BEA resin was formed in the cellulose matrix, without changing the chemical structure. The as-prepared films maintain the merits of cellulose (transparency, dimensional stability, etc.), and the method presented here has the potential for improving the waterproof properties of cellulose.

## 2. Results

**Figure 1:**
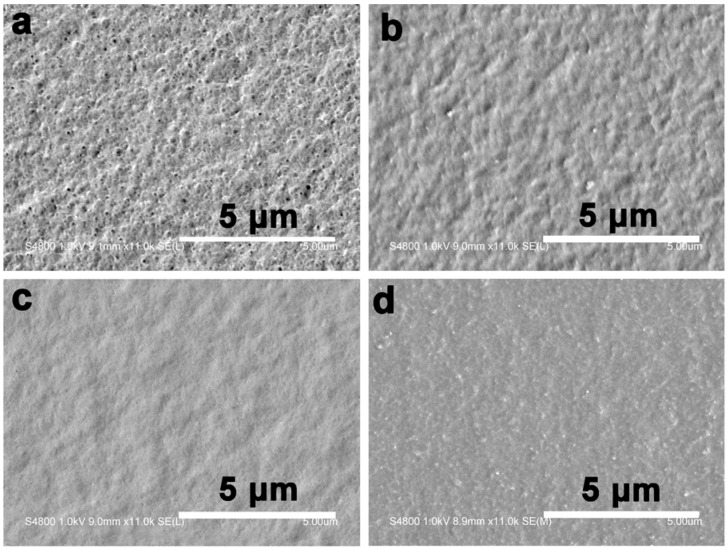
Scanning electron microscopy (SEM) image of the surface morphologies of the composite films. (**a**–**d**) are for RCBEA5, RCBEA15, RCBEA35, and RCBEA50, respectively.

**Figure 2:**
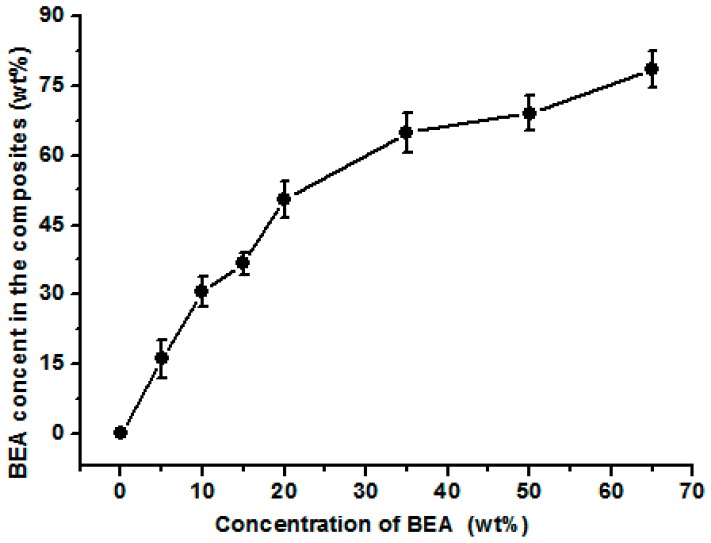
The influence of the concentration of the biphenyl A epoxy acrylate prepolymer on the content of the cured resin in the composite films.

Figure 1 shows the surface morphologies of the composite films. The composite films treated with the prepolymer solution at a concentration less than 10 wt % had a rough surface, indicating the incorporated resin was not enough to fill the pores in the matrix. For the composite films treated with BEA prepolymer concentration higher than 10 wt %, the surface was smoother, implying the influence of the prepolymer concentration on the composite microstructure. The weight of the resin incorporated in the films increased with increasing prepolymer concentration (Figure 2). The BEA prepolymer concentration was directly proportional to the amount of BEA resin absorbed. The unique structure resulted from the phase separation of the cellulose solution during regeneration, where solvent-rich regions contributed to the pore formation. The immersion of the porous structured cellulose film in the BEA prepolymer solution facilitated the penetration of the prepolymer into the scaffold through the pores. After exposure to UV light, the BEA prepolymers were cross-linked directly with the cellulose matrix, leading to the formation of the new network consisting of BEA resin. The porous structured cellulose matrix was a novel scaffold to incorporate/absorb prepolymer. The resin content in the matrix could be controlled by varying the BEA prepolymer concentration, which is very important for the property control of the cellulose-based composites.

**Figure 3:**
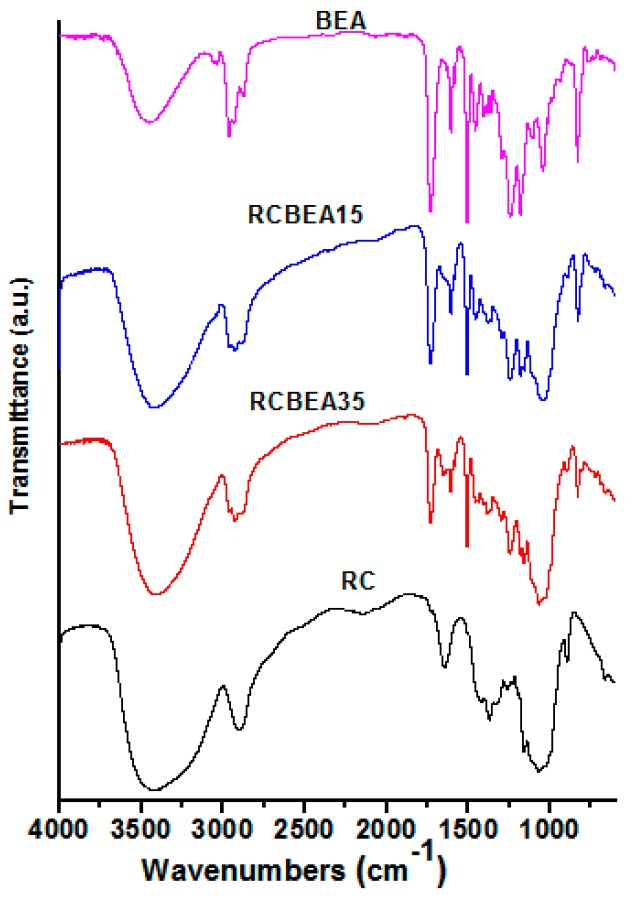
Fourier-transform infrared (FT-IR) spectra of the RC film, biphenyl A epoxy acrylate resin, and some composite films.

The incorporation of BEA resin was further verified by the Fourier-transform infrared (FT-IR) test (Figure 3). An obvious difference in the regenerated cellulose (RC) and BEA was observed in the 3200–3600 cm^−1^ region with a broad O–H stretch band of RC, indicating the complexity of hydrogen bond networks in RC and water molecules in the amorphous region. The peak weakened or disappeared at 1650 cm^−1^, which was assigned to the water in cellulose, suggesting the improvement of the composite films in hydrophobicity. The strong bands near 2900 cm^−1^ were due to the C–H stretch vibrations. However, for the composite films, the peaks shifted to higher wavenumbers, and the shoulder peaks in the lower (2870 cm^−1^) and higher (2940 cm^−1^) energy sides of the C–H peaks were ascribed to the symmetric and asymmetric -CH_2_ stretch vibrations of the BEA, respectively, indicating no chemical interaction between cellulose and BEA. The pure resin showed several sharp peaks at 1450–1604 cm^−1^, which were typical of the benzene ring vibration. The characteristic peaks in the spectra of pristine resin and cellulose were observed in the spectra of the composites, demonstrating the presence of the combined chemical behavior of both materials in the composites.

**Figure 4:**
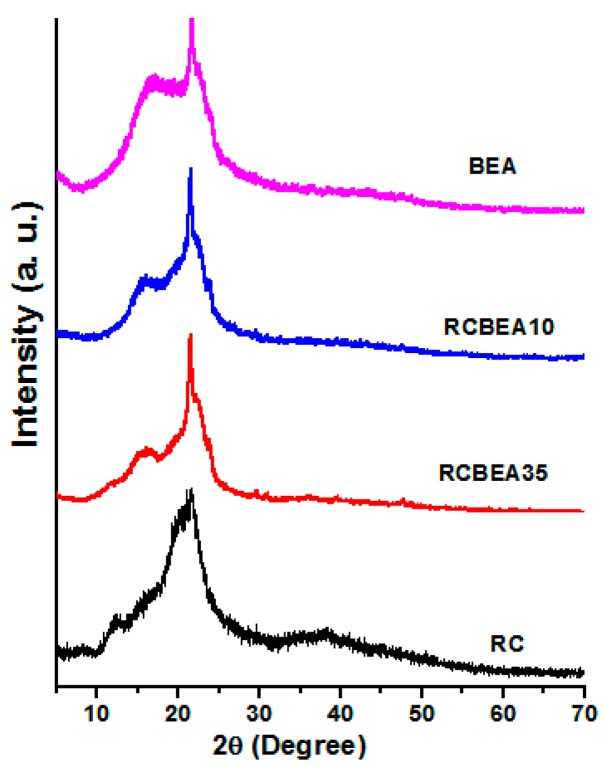
Wide angle X-ray diffraction (WAXD) patterns of the regenerated cellulose (RC) film, bisphenol A epoxy acrylate resin, and some composite films.

**Figure 5:**
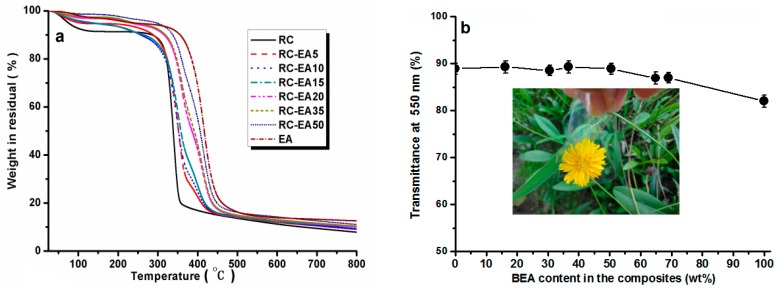
Thermal analysis of the RC, BEA, and composite films with different resin content (**a**); and optical transmittance at 550 nm of the RC, BEA, and composite films (**b**); the insert was the photo of the RCBEA35.

Figure 4 shows the X-ray diffraction (XRD) patterns of the RC, BEA, and composite films. The pure cellulose film showed the main diffraction peaks at 2θ = 12.3°, 20.3°, and 21.7°, which were ascribed to the typical cellulose II. For the BEA, there were only two large peaks in the 2θ ranging from 17° to 21.7°. The patterns were similar for the composite films, and the diffraction peaks of both cellulose and BEA were overlapped in the composites, further confirming the successful incorporation of the resin in the cellulose matrix. Figure 5a shows the thermal stability of the composites in N_2_. All the samples showed a heat-resistance up to 300 °C. A ~8% weight loss for RC around 100 °C was ascribed to the release of moisture from the samples, while for the composites, the weight loss was lower than 5% in the same temperature region, indicating that the loading of BEA resin increased the hydrophobicity of the composites. With increasing temperature, the RC film showed an obvious weight loss slope within the temperature range of 300–350 °C, which resulted from the decomposition of cellulose. Pure BEA resin had better thermal stability than RC film, and the loaded BEA contributed to the thermal stability of the composites, resulting in a higher thermal stability in the composites than in the RC, and the stability increased with increasing BEA resin. It is worth noting that the thermal stability of the composite films was higher than that of the synthetic polymers commonly used in photoelectric devices, such as polyethylene terephthalate (PET). Figure 5b displays the light transmittance of the RC, BEA, and composite films within the wavelength range of 200–800 nm. The transmittance of the RC film was about 88% at 550 nm, which was higher than that of the pure BEA resin. The loading of the BEA resin changed the transmittance of the composite films slightly, but it was still higher than that of BEA resin. As is well known, the optical property of a transparent polymer composite is usually expressed by the following formula [22]:

(1) $$\frac{I}{I_{0}}\equiv \exp\left[-\frac{V_{p}\chi r^{3}}{4\lambda^{4}}\left(\frac{n_{p}}{n_{m}}-1\right)\right]$$

where χ is the optical path length, *V_p_* is the filler volume fraction, *r* is the filler radius, λ is the light wavelength, and *n_p_* and *n_m_* are the refractive indices of the fillers and matrix, respectively. The refractive indexes of cellulose and BEA are about 1.54 and 1.56, respectively, suggesting that the difference in the refractive index of the resin and cellulose matrix can be neglected. The composite films with a higher content of BEA resin showed poor light transmittance, which probably resulted from the light refraction and reflection at the cellulose/BEA interfaces. It has also been reported that the transmittance of the cellulose acetate/epoxy resin composite films decreased with increasing epoxy resin content. Moreover, the surface reflection might also decrease their transmittance due to the difference in refractive index (RI). The relationship between light reflection and RI can be described by the following equation.

(2) $$\Gamma ={\left[(n_{R}-n_{F})/(n_{R}+n_{F})\right]}^{2}$$

where Γ is the reflective coefficient, and *n_R_* and *n_F_* are RI of BEA and cellulose, respectively. The higher the difference in RI, the higher the reflective coefficient was for the composites. With BEA resin treatment, the cellulose/air interfaces were replaced with three different interfaces, cellulose/BEA resin, cellulose/air, and air/BEA resin in the interior structure of the composite films. FT-IR results indicated that no chemical reaction occurred between cellulose and BEA, and the increased interfaces would decrease the transmittance of the composite films.

**Figure 6:**
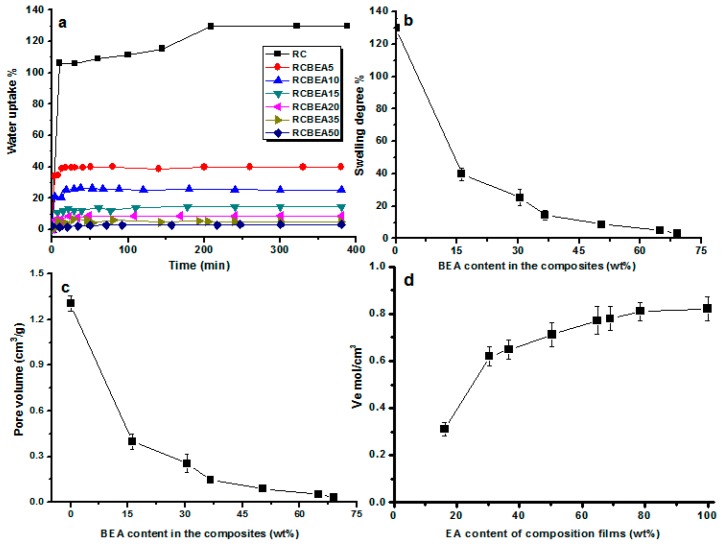
Water uptake ability of the films with a different content of loaded resin (**a**); and the effects of epoxy resin content on the swelling degree of the films (**b**); volume of pores in the films estimated with the uptake of water as a function of the resin content (**c**); and the degree of cross-link (*V_e_*) with the composite films as a function of the resin content (**d**).

Figure 6a shows water uptake ability of the films as a function of the immersion time. All the materials absorbed water in a similar pattern, and after about 20 min of relatively fast uptake, the sorption slowed down and reached a plateau gradually. The composite films obviously absorbed less water than that of the pure RC film. RC film had a swelling degree of about 129%, which often limits its usage. The swelling degree decreased to 40% for the composite film containing 16 wt % BEA, and the swelling degree of the composites decreased with increasing BEA resin content (Figure 6b). Based on its molecular structure, BEA resin has a hydrophobic characteristic, which favors the hydrophobic performance of the composites. Additionally, the cross-linked BEA resin in the cellulose matrix would reduce the water absorption and block the movement of the molecular chain during swelling, thus decreasing the swelling degree caused by water for the cellulose composite films. Figure 6c shows that the static free volume decreased with an increase in the mass fraction of BEA resin, where the total volume of pores (*V_p_*) of the composite films against the BEA resin content was plotted. The *V_p_* of pure cellulose film was about 1.3 cm^3^·g^−1^. The incorporated BEA resin had a notable influence on the *V_p_* of the composites. For the composite films containing about 16 wt % BEA, the *V_p_* decreased to about 0.39 cm^3^·g^−1^, and with increasing BEA concentration, the *V_p_* of the composite films decreased and reached a plateau gradually. The composite films tended to swell in the same pattern, and the equilibrium-swelling ratio (*Q*) and *V_p_* values were slightly lower, indicating the formation of numerous effective cross-links in the resin, which imposed a restriction on the swelling capacity of the materials, especially at a higher BEA concentration.

**Figure 7:**
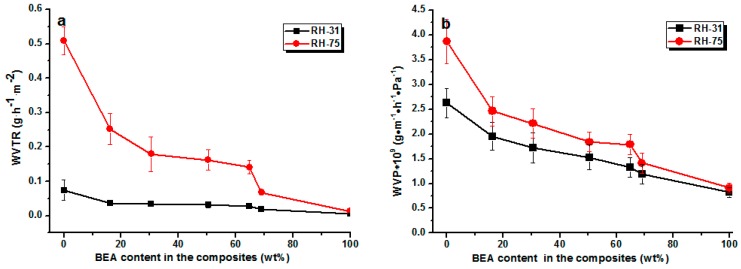
Water vapor transmission rate (WVTR) (**a**) and water vapor permeability (WVP) values (**b**) of the composite films with a different content of loaded resin at a different relative humidity.

The water vapor permeability of the composite films was also low (Figure 7). As expected, the loading of BEA resin reduced the permeability towards water vapor at all relative humidities. The water vapor permeability (WVP) value of the RC film was about 3.9 × 10^−9^ g·m^−1^·h^−1^·Pa^−1^, while that of the RCBEA5 with 16% resin was 2.48 × 10^−9^ g·m^−1^·h^−1^·Pa^−1^ at 75% humidity, which was about a 37% reduction, confirming the increase in hydrophobicity after the modification. The permeability of RC and composite films towards water vapor decreased with decreasing relative humidity, which was consistent with previous reports for other polysaccharides (starches) [23]. As indicated by the water sorption isotherms, the penetration of water through hydrophilic polymers is a complex process and is determined by the amount of water absorbed. It has been reported that at low relative humidity (<40%), the water molecules are absorbed in the amorphous phase of the hydrogen bond active sites, but at higher relative humidity (>70%), the water molecules are accumulated rather than absorbed on the active sites, leading to a drastic increase of the solubility.

**Figure 8:**
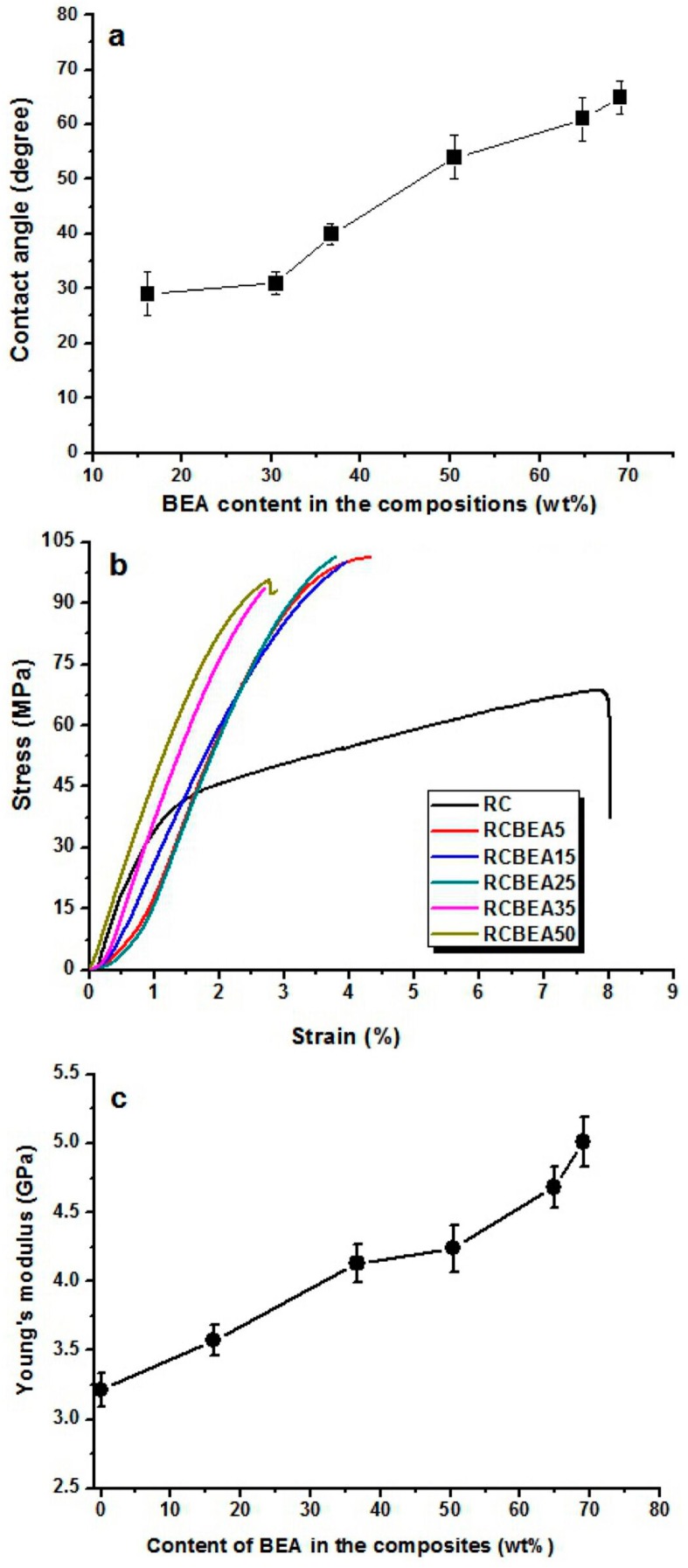
Contact angle images of water droplets on RC and composite films (**a**); and the stress-strain curves of the RC and composite films (**b**); and the corresponding Young’s modulus values of the films (**c**).

Figure 8a shows the average water contact angle of the RC and composite films. Once dripped on the surface of the RC film, the water was absorbed by the RC film within 2 min, making it difficult to determine the contact angle value of the RC film. However, for the composite films treated with BEA resin, the water volume almost remained unchanged on their surfaces during the test, indicating that no water absorption occurred in the composite films. In the process of preparation, the porous structured cellulose films first absorbed BEA prepolymer and acetone solvent, and after the evaporation of acetone, the cellulose matrix would shrink and the BEA prepolymer would be incorporated into the pores or deposited on the surface of the cellulose scaffold. When exposed to UV light, the BEA prepolymer would be cross-linked and BEA resin would be synthesized homogeneously in the cellulose matrix, leading to a smooth surface in the composite films, as indicated in the SEM results. With the BEA resin content increased from 16 to 70 wt %, the water contact angle of the composite films increased from 29° to 65°, indicating a remarkable improvement in hydrophobicity. Based on the results, there was a large amount of BEA resin in the cellulose matrix (Figure 2). The BEA resin with a low surface energy could improve the hydrophobicity by trapping air, whose water contact angle is believed to be 180°.

The mechanical behavior of the RC and composite films was investigated by using a tensile test at room temperature. Figure 8b shows the stress-strain curves of the samples. The RC film had a tensile strength of about 68 MPa, with a break elongation of about 8%. With the treatment of the resin, the tensile strength was increased obviously. For the RCBEA-5 composite film containing about 16 wt % resin, the tensile strength was about 101 MPa, a 48% increase over that of RC film, and the break elongation was about 4.3%. The decrease in the break elongation was ascribed to the molecular structures of the biphenyl A epoxy acrylate prepolymer and the cellulose. Cellulose has a semi-rigid molecular chain and strong inter- and intra-molecular hydrogen interaction, and the biphenyl A epoxy acrylate resin itself also has strong interaction. The pure biphenyl A epoxy acrylate resin has a poor elongation at break, which is lower than that of cellulose. Another factor was the formed double network in the composite films. Therefore, the break elongation was decreased. With increasing resin, the tensile strength changed slightly, but the elongation decreased from 4.3% for RCBEA-5 to 2.7% for RCBEA-50. The Young’s modulus increased from 3.2 GPa for RC film to 5.1 GPa for RCBEA50. The significant increase in the Young’s modulus value of the composites was mainly attributed to the formed double network structure, which could be responsible for the unusual reinforcing effect.

## 3. Materials and Methods

### 3.1. Materials

Native cellulose with the viscosity-average molecular weight (*M_η_*) of 1.07 × 10^5^ in cadoxen solution at 25 °C (Cotton linter, α-cellulose ≥ 95%) was purchased from Hubei Chemical Fiber Co. Ltd. (Xiangfan, China). Biphenyl A epoxy acrylate prepolymer (BEA) was provided by Pengbo Co. Ltd. (Shanghai, China).

### 3.2. Preparation of BEA/Cellulose Composite Film

Native cellulose was dissolved into LiOH/urea solution (4.6 wt %/15.0 wt %) with a concentration of 5 wt %. The bubble-free solution was poured on a glass plate with the solution thickness controlled within 1 mm. Next, the plate was bathed in anhydrous ethanol solution for 10 min. After washing with deionized water, the RC films were treated with acetone to remove the water, and then stored in absolute acetone for further use.

The composite films were prepared through a solution impregnation process. Briefly, after replacing the water in the cellulose hydrogel films with acetone, the obtained RC films were immersed in freshly prepared BEA prepolymers (acetone was used as solvent) and curing agent (acetone was used as solvent) solution for 8 h to incorporate the prepolymers into the cellulose scaffold. After evaporation of some acetone, the cellulose films were exposed to UV light and cured for 5 min, then were washed first with acetone and then with water, and finally were dried at room temperature. The composite films were treated at BEA prepolymer concentrations of 5, 10, 15, 25, 35, and 50 wt %, and designated as RCBEA5, RCBEA10, RCBEA15, RCBEA25, RCBEA35, and RCBEA50, respectively. Pure cellulose and BEA films were defined as RC and BEA, respectively.

### 3.3. Characterization

The samples were mixed with KBr and ground into powders, and then pressed to form a sample disk for the Fourier-transform infrared (FT-IR) spectroscopy (FT-IR 615, Jasco, Tokyo, Japan) test. Wide-angle X-ray diffraction (XRD) tests were performed with a XRD diffractometer (D8-Advance, Bruker, Aubrey, TX, USA). Cu Kα radiation (the weighted average λ is 0.15406 nm) was used at 40 kV and 40 mA, and the diffraction angles were recorded in the region of 2θ from 8° to 70°. Thermal gravimetric analysis (TGA) was carried out on a thermogravimetric analyzer (TGA/DSC1/1100SF, Mettler Toledo, Columbus, OH, USA). Scanning electron microscopy (SEM) tests were performed with a Hitachi S4800 (Tokyo, Japan) microscope. The water contact angle of the composite films was determined by using a contact angle analyzer (DCA315, San Diego, CA, USA). The mechanical property test of the films was carried out on a universal tensile tester (CMT 6503, Shenzhen SANS Test Machine Co. Ltd., Shenzhen, China) with a speed of 2 mm∙min^−1^ at room temperature.

The *Q* of the films was estimated through a mass balance. A piece of film with a size about 4 cm × 4 cm was immersed into distilled water until it reached swelling-equilibrium state, and then removed from the solution. The water on its surface was wiped off, and then the weight of the wet film was measured. After that, the film was dried in a vacuum oven at 70 °C for about 48 h and then weighed again. The *Q* was calculated by the following formula:

(3) $$Q=\frac{W_{h}-W_{d}}{W_{d}}$$

where *W_h_* is the equilibrium weight of the swollen sample and *W_d_* is the weight of the dried sample. Five replicates were performed to obtain an average value of *Q*.

Water vapor transmission rate (WVTR) of the films was measured according to the method based on the ASTM E96-95-standard for water vapor transmission of materials. Different relative humidity (RH) environments were established inside the test chamber using saturated aqueous solutions of different salts: magnesium chloride (31% of RH at 25 °C); sodium chloride (75% of RH at 30 °C). For the test, glass cups with a diameter of 3 cm and a depth of 4 cm were used. To maintain 0% RH in the cup headspace, 2 g of dried CaCl_2_ was added to the cup, a film was sealed over the rims of the cups with molten paraffin, and then the cups were placed in hermetically sealed jars held at 30 °C and 75% RH (saturated NaCl solution was used). To maintain RH, 1000 mL of water was placed in the bottom of the jar. The weight of the cups was measured every 1 h for 3 days, and the amount of the water permeated through the films was determined by the added weight of the cups. *WVTR* and water vapor permeability (*WVP*) were calculated according to the following formula [24]:

(4) $$\begin{array}{l} WVTR=\frac{\Delta w}{\Delta t\times A}\\ WVP=\frac{WVTR\times L}{\Delta p} \end{array}$$

where *WVTR* was in g/s·m^2^, △*w*/△*t* was the rate of water gain in s/h, *A* was the exposed area of the film in m^2^, *L* was the mean thickness of samples in m, and △*p* was the difference in partial water vapor pressure between the two sides of film samples in Pa. The water vapor pressure on the high-stream side of the film was 3.2 kPa (water vapor pressure of saturated NaCl aqueous solution at 30 °C), while the low-stream side was assumed to be zero.

The *V_p_* of the films was obtained through the water uptake behavior. As a non-solvent for cellulose and BEA, *V_p_* was calculated from the following formula [25]:

(5) $$V_{p}=\frac{M_{\mathrm{wet}}-M_{\mathrm{dry}}}{\rho \times M_{\mathrm{dry}}}$$

where *M*_wet_ was the weight of the samples immersed in water until it reached swelling-equilibrium. ρ was the density of water (0.995 g·mL^−1^, 30 °C).

On the basis of Flory-Rehner theory [26,27,28], the cross-link density (*V_e_*) of the films was determined from the equilibrium swelling measurements at room temperature:

(6) Ve=−ln(1−ϕr)+ϕr+Xϕr2V1(ϕr13−ϕr2)

(7) $$\phi_{r}=\frac{\frac{W_{d}-W_{ins}}{\rho_{r}}}{\frac{W_{d}-W_{ins}}{\rho_{r}}+\frac{W_{sol}}{\rho_{s}}}$$

where *V_e_* was the network chain density (mol·cm^−3^), *X* was the Flory-Huggins interaction parameter (0.391), *V*_1_ was the molar volume of acetone (75.76 cm^3^·mol^−1^), φ*_r_* was the BEA volume fraction, and *w_d_* and *w_ins_* were the deswollen and initial weights of the samples, respectively. ρ*_r_* and ρ*_s_* were the density of cured polyurethane acrylate (1.051 g·cm^−3^) and acetone (0.767 g·cm^−3^), respectively, and *w_sol_* was the acetone weight after 72 h of uptake. The values reported were the average of at least three test pieces per sample (~20 mm diameter).

## 4. Conclusions

Cellulose-based composite films were prepared via dipping BEA prepolymers into cellulose matrix and then by UV exposure. The BEA resin was cross-linked in the cellulose scaffold and a double network structured composite film was obtained. The incorporated resin contributed to the change in the microstructure of the cellulose matrix, leading to a decrease not only in the swelling degree of the composites but also in the water uptake and the permeability towards water vapor gas at a different relative humidity. Additionally, measurements of contact angle with water demonstrated that the hydrophobic properties of the composite films were obviously improved. Furthermore, the loading of the resin also enhanced the mechanical properties of the films, with the Young’s modulus value increased from 3.2 GPa of the RC film to 5.1 GPa of RCBEA50. The composite films also showed good optical transmittance, and enhanced thermal tensile strength. The overall data indicate that the composite films have combined the synergetic advantages of cellulose and BEA resin, and have potential applications as high-performance packaging materials.
